# Enhanced Therapeutic Effects of Human iPS Cell Derived-Cardiomyocyte by Combined Cell-Sheets with Omental Flap Technique in Porcine Ischemic Cardiomyopathy Model

**DOI:** 10.1038/s41598-017-08869-z

**Published:** 2017-08-18

**Authors:** Masashi Kawamura, Shigeru Miyagawa, Satsuki Fukushima, Atsuhiro Saito, Kenji Miki, Shunsuke Funakoshi, Yoshinori Yoshida, Shinya Yamanaka, Tatsuya Shimizu, Teruo Okano, Takashi Daimon, Koichi Toda, Yoshiki Sawa

**Affiliations:** 1 Department of Cardiovascular Surgery, Osaka University Graduate School of Medicine, Suita, Japan; 2 Medical Center for Translational Research, Osaka University Hospital, Suita, Japan; 3 Center for iPS cell Research and Application, Kyoto University, Kyoto, Japan; 4 Institute of Advanced Biomedical Engineering and Science, Tokyo Women’s Medical University, Tokyo, Japan; 5 Department of Biostatistics, Hyogo College of Medicine, Nishinomiya, Japan

## Abstract

Transplant of human induced pluripotent stem cell derived cardiomyocytes (hiPS-CMs) cell-sheet is a promising approach for treating ischemic cardiomyopathy (ICM). However, poor blood supply to the transplanted cell-sheet is a concern related to the effectiveness and durability of the treatment. Herein, we hypothesized that the combined the omentum flap might enhance survival and the therapeutic effects of hiPS-CM cell-sheet transplant for ICM treatment. Treatment by Wnt signaling molecules in hiPS cells produced hiPS-CMs, which were magnetically labeled by superparamagnetic iron oxide (SPIO), followed by culture in the thermoresponsive dishes to generate hiPS-CMs cell-sheets. A porcine ICM model included 4 groups; sham operation, omentum flap only, cell-sheet only, or combination therapy. Ejection fraction (EF) was significantly greater in the cell-sheet only and combination group compared to the other groups during the follow-up period. At 3 months, the EF of the combination group was significantly greater than that of the cell-sheet only group. Consistently, the survival rate of the SPIO-labeled hiPS-CMs, as assessed by MRI, was significantly greater in the combination group than in the cell-sheet only group. This cell delivery system would be useful in optimizing the hiPS-CM cell-sheet transplant for treating severe heart failure.

## Introduction

Stem cell therapy has recently emerged for treating heart failure, and numerous preclinical and clinical studies using various types of stem cells have been proven to improve cardiac functions and attenuate left ventricular remodeling^1–3^. However, the ideal cell type or the optimum cell delivery method is still unknown^1–3^. We have demonstrated that advantages of cell-sheet technique as a cell delivery method in stem cell therapy for the treatment of heart failure^4^. This technique preserves extra cellular matrix without artificial scaffolds, which may prevent cell detachment -associated “anoikis”^5^. In contrast to the myocardial needle injection, the cell-sheet technique can deliver a large number of cells to failed heart with high retention rate of transplanted cells and minimal injury to the host myocardium^6, 7^.

Human induced pluripotent stem (hiPS) cells, which have a capacity of unlimited proliferation and differentiation to cardiomyocyte^8, 9^, are promising cell source for myocardial regeneration therapy^10^. We have explored a new strategy of myocardial regeneration therapy using hiPS cells and cell-sheet technique to aim a more effective stem cell therapy for heart failure. We demonstrated the feasibility and therapeutic efficacy of transplantation of human iPS-derived cardiomyocytes (hiPS-CMs) sheet for a porcine ischemic cardiomyopathy model^11^, however, long-term engraftment of transplanted cells has remained to be concerned^11^. This poor engraftment of the transplanted cells is considered to be resulted from ischemia caused by poor vascularization of the transplanted sites and inflammation with attendant oxidative stress and release of cytotoxic cytokines^1–3^. To overcome the issue of long-term engraftment of transplanted cells, we have focused on the omentum, because the omentum is known to be a vascular-rich organ, contain abundant angiogenic factors, and have anti-inflammatory effects^12^. We have expected the omentum as a blood supply source, and reported that combination of the pedicle omentum flap with cell-sheet enhanced the survival of transplanted hiPS-CMs in an uninjured porcine heart^13^.

Herein, we hypothesized that the pedicle omentum flap technique may enhance survival of hiPS-CMs and the therapeutic capacity of hiPS-CM sheet transplant in a porcine ischemic cardiomyopathy model. In this study, we compared survival of hiPS-CMs after transplantation in a diseased heart, with or without the pedicle omentum flap, and we also investigated whether improvement of cardiac functions increased by the additive omentum flap compared with the hiPS-CM sheet itself in a porcine cardiomyopathy model.

## Results

### Cardiomyogenic differentiation of hiPS cells and cell-sheet generation

**Figure 1:**
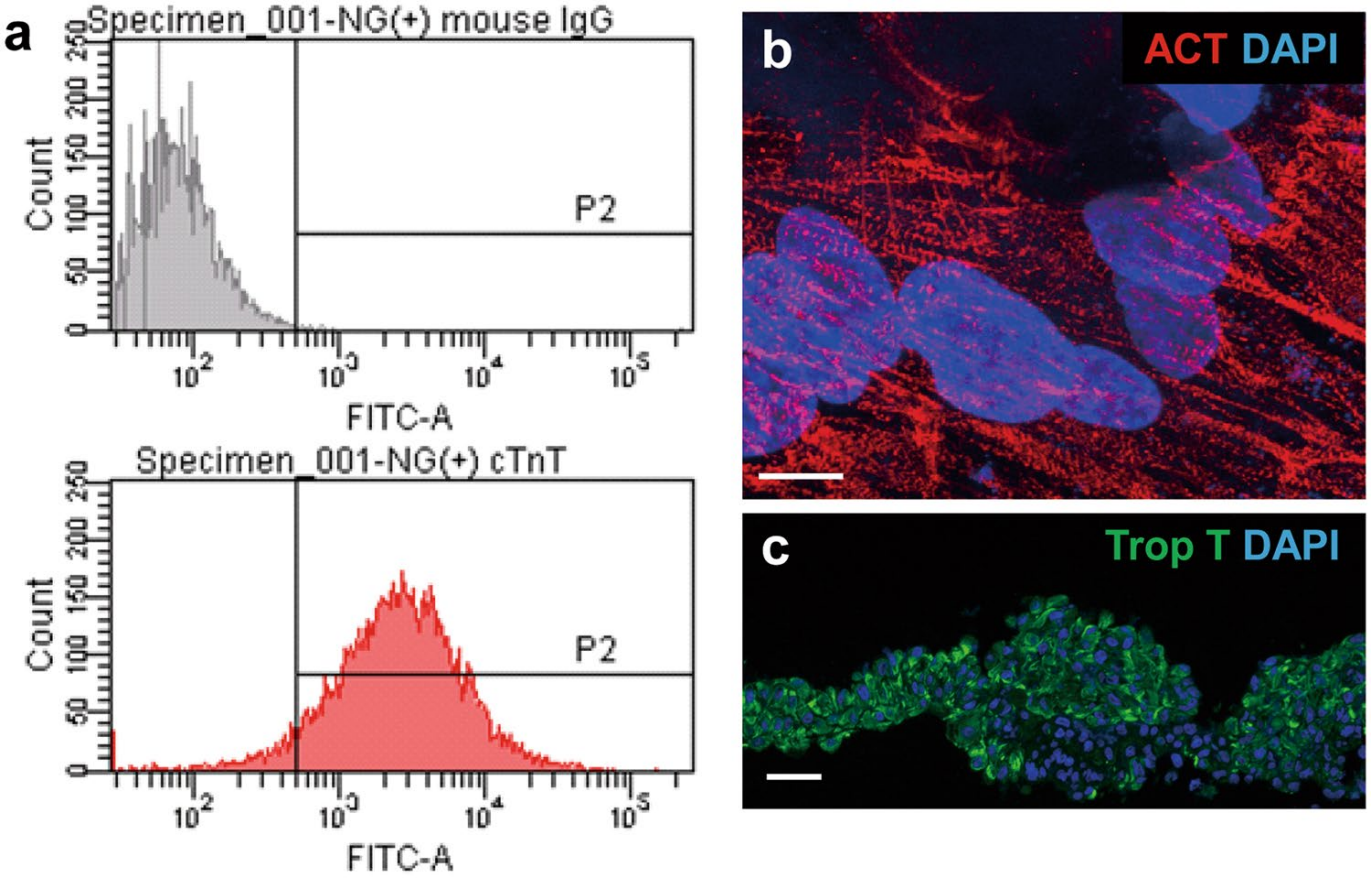
Characterization of hiPS-CMs and hiPS-CM cell sheet. (**a**) Expression of cardiac troponin T (cTNT) after differentiation and purification of hiPS-CMs was determined by flow cytometry anaysis. (**b**) After differentiation and purification, sarcomere structure was visualized by sarcomeric alpha actinin staining in hiPS-CM. (**c**) Immunostaining of the hiPS-CM cell sheet with cTNT antibody (green). The cell nuclei were counterstained with 4′,6-diamidino-2-phenylindole (DAPI; blue). Scale bar, 10 μm in (**b**) and 100 μm in (**c**).

Differentiation of hiPS cells into cardiomyocytes was induced by treatment of the embryoid bodies formed from cultured hiPS cells with Wnt3a and R-spondin-1 in thermoresponsive dishes (10-cm Upcell dishes). Subsequently, the differentiated hiPS cells were purified by culture in glucose-free medium to yield 1–2 × 10^7^ hiPS-CMs. Approximately 80% (84.6 ± 6.8%) of the hiPS-CMs were positive for cardiac troponin T (cTNT), as determined by flow cytometry (Fig. 1a), and evidence of sarcomeres among the hiPS-CMs was demonstrated by immunocytochemistry with an anti-sarcomeric alpha actinin antibody (Fig. 1b). Human mesenchymal stem cells (hMSCs) are known to have the potential to induce immunologic tolerance^14^ and enhance the structural characteristics of engineered tissue^15, 16^. Therefore, to fill the cell-free space in the Upcell dishes and to aid in lifting up the cell sheets, we added hMSCs to the hiPS-CM culture, and incubated the dishes at room temperature, which induced spontaneous detachment of the cells into scaffold-free hiPS-CM cell sheets. Immunohistolabeling showed that the large number of cells in the hiPS-CM cell sheets were homogeneously positive for cTNT (Fig. 1c).

### Functional recovery in a porcine ICM model after the treatment assessed by serial CMR

**Figure 2:**
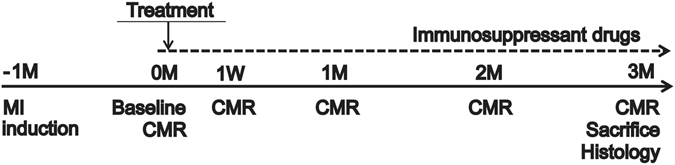
Study protocol of the mini-pig experiments. Schedule of cardiac magnetic resonance imaging (CMR) and histological evaluations.

**Figure 3:**
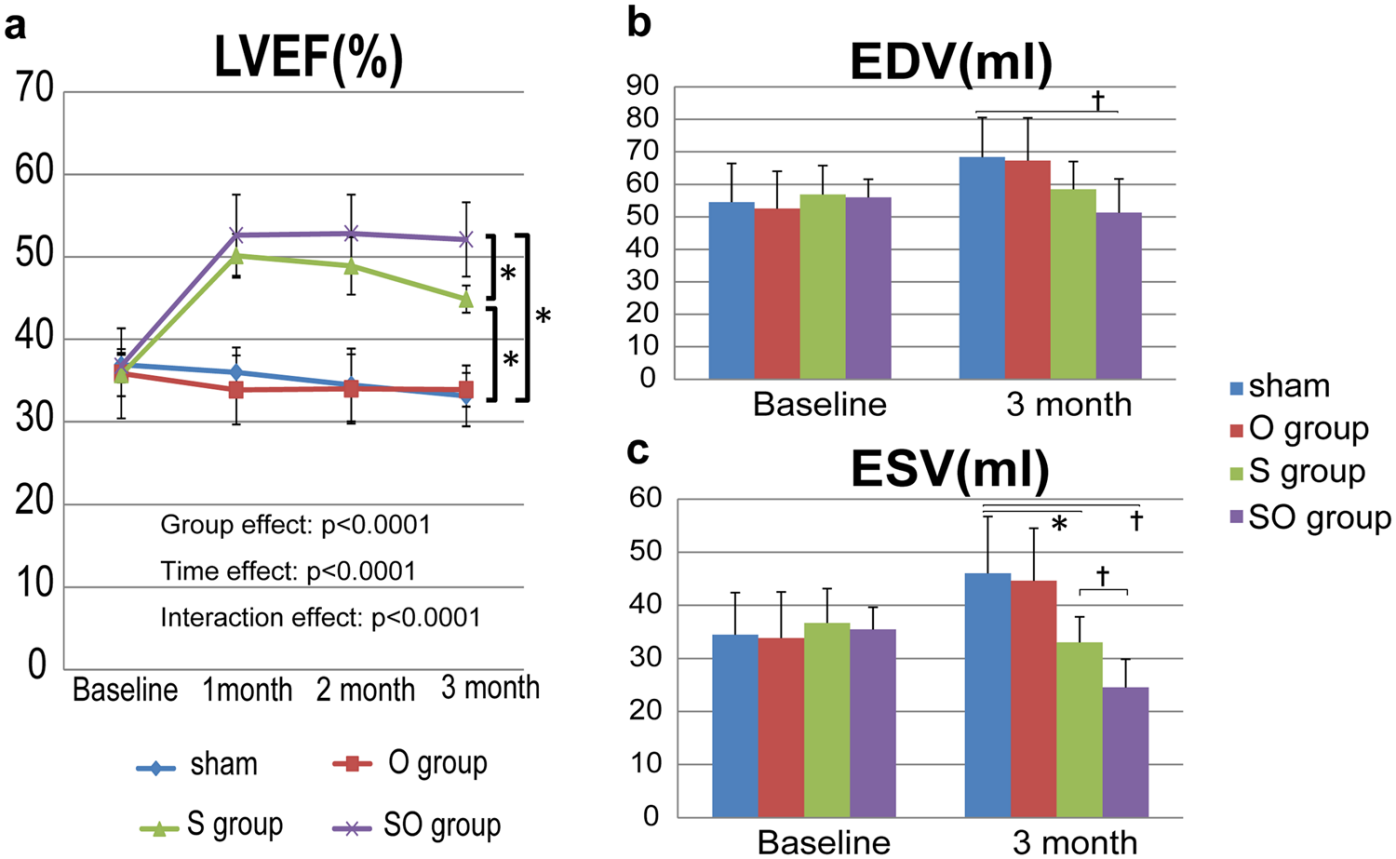
CMR analysis for global cardiac functions: LVEF in a, LVEDV in b, and LVESV in c. (**a**) LVEF at 3 months was significantly greater in the SO group than in the S group. (**b**) LVEDV at 3 months in combination group showed significantly smaller than those in the sham group. (**c**) LVESV at 3 month in the S and SO group showed significantly smaller than that in the sham group. LVESV at 3 months were significantly smaller in the SO group than in the S group. ^†^p < 0.05, *p < 0.01.

We established a porcine ICM model by placement of an ameroid constrictor (COR-2.50-SS, Research Instruments) around the left anterior descending coronary artery in mini-pigs (Japan Farm) through a left thoracotomy^17^. Four weeks after MI induction, we treated them via median sternotomy under general anesthesia. All animals were immunosuppressed by daily administration of tacrolimus (0.75 mg/kg, Astellas), mycophenolate mofetil (500 mg, Teva Chech Industries s.r.o,), and predonisolone (20 mg, Takeda Pharmaceutical Co Ltd.) daily from 5 days before the treatments until sacrifice. The experimental workflow was shown in Fig. 2. The treatments included 4 options, and 23 mini-pigs with MI were randomly divided into 4 treatment groups: cell-sheets with the omentum flap (SO group = 7), cell-sheet only (S group = 6), the omentum flap only (O group = 5), or sham operation (Sham group = 5). We prepared hiPS-CM sheet, as previously described^11, 13, 18^. The hiPS-CMs were labeled with the superparamagnetic iron oxide (SPIO) ferucarbotran (Resovist; Bayer Pharma), by using the hemagglutinating virus of Japan envelope (HVJ-E) vector (GenomeOne-Neo, Ishihara Sangyo)^19, 20^. We successfully performed the treatments, and there was no mortality related to the procedure or otherwise prior to the planned sacrifice. To evaluate cardiac functions after the treatments, we performed serial cardiac magnetic resonance imaging (CMR) on the same mini-pigs prior to (baseline) and 1 month, 2 month, and 3 months after treatments, and evaluated in LVEF, LV end-diastolic volume (LVEDV), and LV end-systolic volume (LVESV). The baseline LVEF, LVEDV, and LVESV did not differ significantly between the 4 groups. The sham operation and the omentum only group showed no improvement in LVEF during follow-up period (LVEF at baseline, 1 M, 2 M, 3 M; Sham group: 36.9 ± 1.2%, 36.0 ± 3.0%, 34.5 ± 4.4%, 33.1 ± 3.7%, O group: 35.9 ± 5.5%, 33.9 ± 4.2%, 34.0 ± 4.2%, 33.9 ± 2.0%, Fig. 3a). The sheet only group and combination therapy showed improvement in LVEF compared with other groups (LVEF at baseline, 1 M, 2 M, 3 M; S group: 35.7 ± 2.6%, 50.1 ± 2.7%, 48.9 ± 3.5%, 44.9 ± 1.6%, SO group: 36.8 ± 2.0%, 52.6 ± 4.9%, 52.8 ± 4.7%, 52.1 ± 4.5%, p < 0.0001 for interaction effect of time and group in the repeated ANOVA, Fig. 3a). Subsequently, improvement in LVEF remained in the combination therapy over 3 months, in contrast, LVEF gradually reduced in the sheet only group in the meantime. LVEF at 3 months was significantly greater in the combination therapy than in the sheet only group (52.1 ± 4.5% versus 44.9 ± 1.6%, P < 0.01, Fig. 3a). In addition, LVEDV at 3 months in combination group showed significantly smaller than those in the sham group (51.3 ± 10.3 ml versus 68.4 ± 12.1 ml, p < 0.05, Fig. 3b). LVESV at 3 month in the sheet only and the combination therapy showed significantly smaller than that in the sham group (Fig. 3c), and LVESV at 3 months were significantly smaller in the combination therapy than in the sheet only group (24.5 ± 5.3 ml versus 33.0 ± 4.8 ml, P < 0.05, Fig. 3c).

### Regional wall motions assessed by serial CMR after the treatments

**Figure 4:**
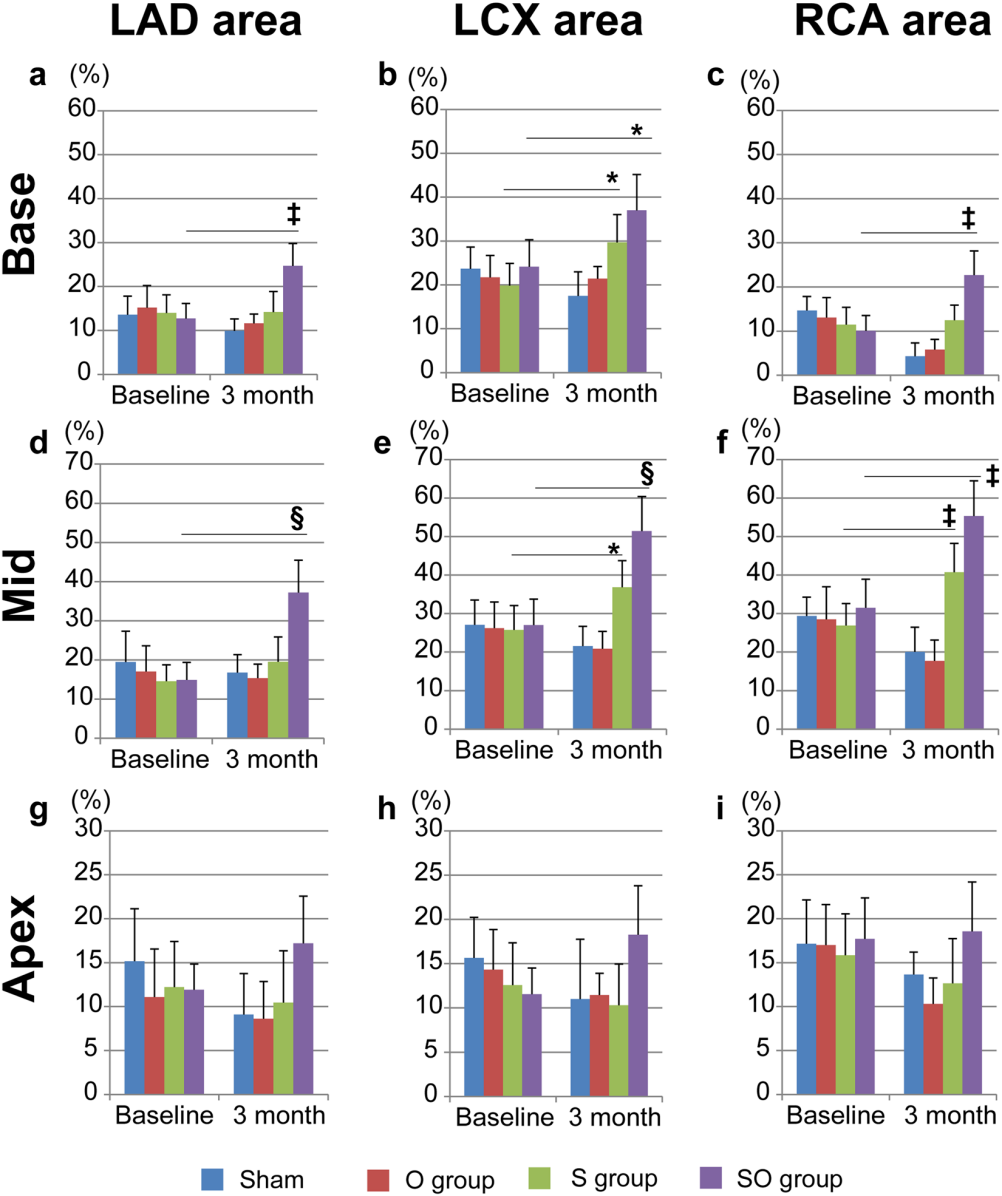
CMR analysis for regional wall motions (**a**–**i**). Regional wall motions at baseline and at 3months after treatments were compared. In the S group, improvements of regional wall motions were demonstrated in 3 regions in (**b**,**e**,**f**). In the SO group, improvements of regional wall motions were observed in 6 regions in (**a**–**f**). Any regional wall motions at apex level had not changed significantly in all treatment groups in (**g**–**i**). ^†^p < 0.05, *p < 0.01, ^‡^p < 0.001, ^§^p < 0.0001 vs. baseline.

The porcine ICM model in the present study had the infarct lesion, the infarct-border lesion, and the infarct-remote lesion. It is an important aspect which lesions the treatment have effects on to investigate underlying mechanisms of the stem cell therapy. We evaluated regional wall motions at baseline and 3 months after the treatments, which obtained by serial CMRs, using the specialized software. The whole heart was divided to 9 regions including the left anterior descending artery (LAD), the left circumflex artery (LCX), and the right coronary artery (RCA) area at each base, mid, and apex level, which were determined referring a standardized LV segmentation model. Any regions in the sham and the omentum only groups were not improved during the follow-up periods. In the sheet only group, improvements of regional wall motions were demonstrated in 3 regions including Base-LCX (20 ± 5% versus 30 ± 6%, p < 0.01), Mid-LCA (26 ± 6% versus 37 ± 7%, p < 0.01) and Mid-RCA area (27 ± 6% versus 41 ± 8%, p < 0.001, Baseline versus 3 months, Fig. 4b,e,f). On the other hand, in the combination therapy, improvements of regional wall motions were observed in 6 regions including Base-LAD (13 ± 4% versus 25 ± 5%, p < 0.001), Base-LCX (24 ± 6% versus 37 ± 8%, p < 0.01), Base-RCA (10 ± 4% versus 23 ± 6%, p < 0.001), Mid-LAD (15 ± 4% versus 37 ± 8%, p < 0.0001), Mid-LCX (27 ± 7% versus 51 ± 9%, p < 0.0001), and Mid-RCA area (32 ± 7% versus 55 ± 9%, p < 0.001, Baseline versus 3 months, Fig. 4a–f). Any regional wall motions at apex level had not changed significantly in all treatment groups (Fig. 4g–i). These suggest that the combination therapy had broad therapeutic effects compared with the sheet only group, but the infarct lesions were not improved.

### *In vivo* analysis of survival of the transplanted SPIO-labeled hiPS-CMs by serial CMR

We also performed serial CMRs to assess the survival of the transplanted SPIO-labeled hiPS-CMs, and tracked intensity of SPIO signal from hiPS-CMs at 1 week (baseline), 1 month, 2 months, and 3 months after the cell transplantation.

**Figure 5:**
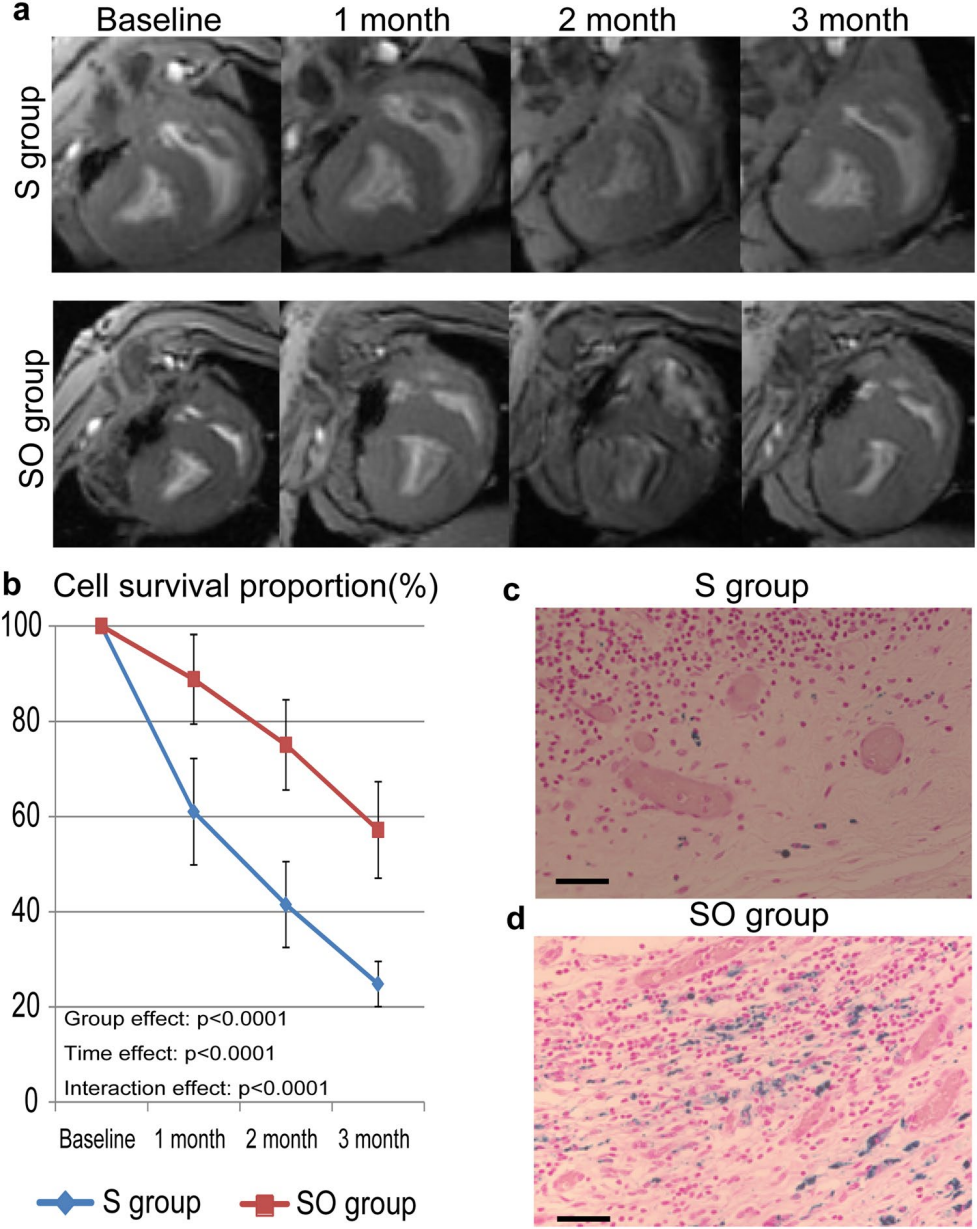
*In vivo* analysis of the survival of SPIO-labeled hiPS-CMs after transplantation. (**a**) Serial CMRs were examined at 1 week (baseline), 1 month, 2 months, and 3 months after SPIO-labeled hiPS-CM cell-sheet transplantation, with or without the omentum. (**b**) Cell survival proportion was estimated by the SPIO-labeled area at 1 month, 2 months and 3 months, corrected by cell survival at 1 week. (**c**,**d**) Cells containing iron, indicative of SPIO-labeled hiPS-CMs, were detected by Prussian Blue staining of sections of the S group (**c**) or the SO group (**d**) at the transplanted area; bar = 50 μm in (**c**,**d**).

SPIO-signals were clearly identified as the hypointense area in the surface of the left ventricle by CMR in all mini-pigs during the study period (Fig. 5a). SPIO-positive hypointense area was gradually decreased in the both groups over the 3 months, while SPIO-positive area was larger and thicker in the combination therapy compared with the sheet only group during the study period. The survival proportion of the SPIO-labeled hiPS-CMs was determined by the formula that the hypointense area at 1, 2 and 3 months after transplantation was divided by the area at 1 week after transplantation as baseline. Both groups showed steady decrease in the cell survival over the 3 months, whereas proportion in the decrease was significantly less in the combination therapy than in the sheet only group at 1 month (89 ± 9% versus 61 ± 11%), 2 months (75 ± 9% versus 42 ± 9%) and 3 months (57 ± 10% versus 25 ± 5%) after the treatment (P < 0.0001 for interaction effect of time and group in the repeated ANOVA, Fig. 5b). Prussian blue staining demonstrated iron-contained cells on the surface of the heart corresponding to the area seen on CMR in both groups. A large number of cells with iron contents were identified in the combination therapy (Fig. 5c,d).

### hiPS-CM sheet with the omentum covering demonstrated increased capillary density, and upregulation of VEGF, SDF-1, and bFGF expression in the transplanted area 3 months after transplant

**Figure 6:**
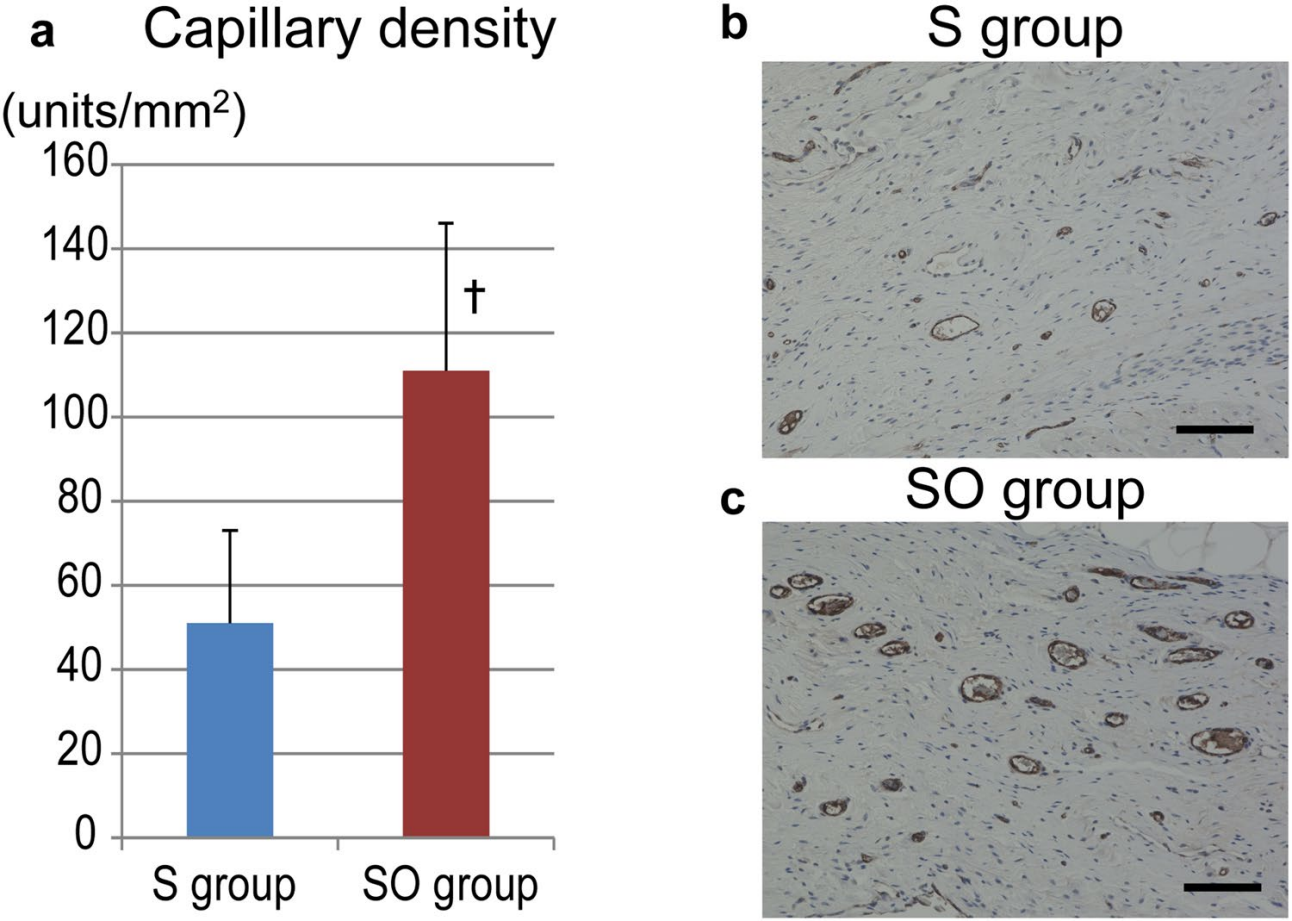
Capillary density in the transplanted area. (**a**) The capillary density in the transplanted area was significantly greater in the SO group than in the S group. Photomicrographs of immunostaining for von Willebrand factor are shown in (**b**,**c**); bar = 100 μm. A. ^†^p < 0.05, vs. the S group.

**Figure 7:**
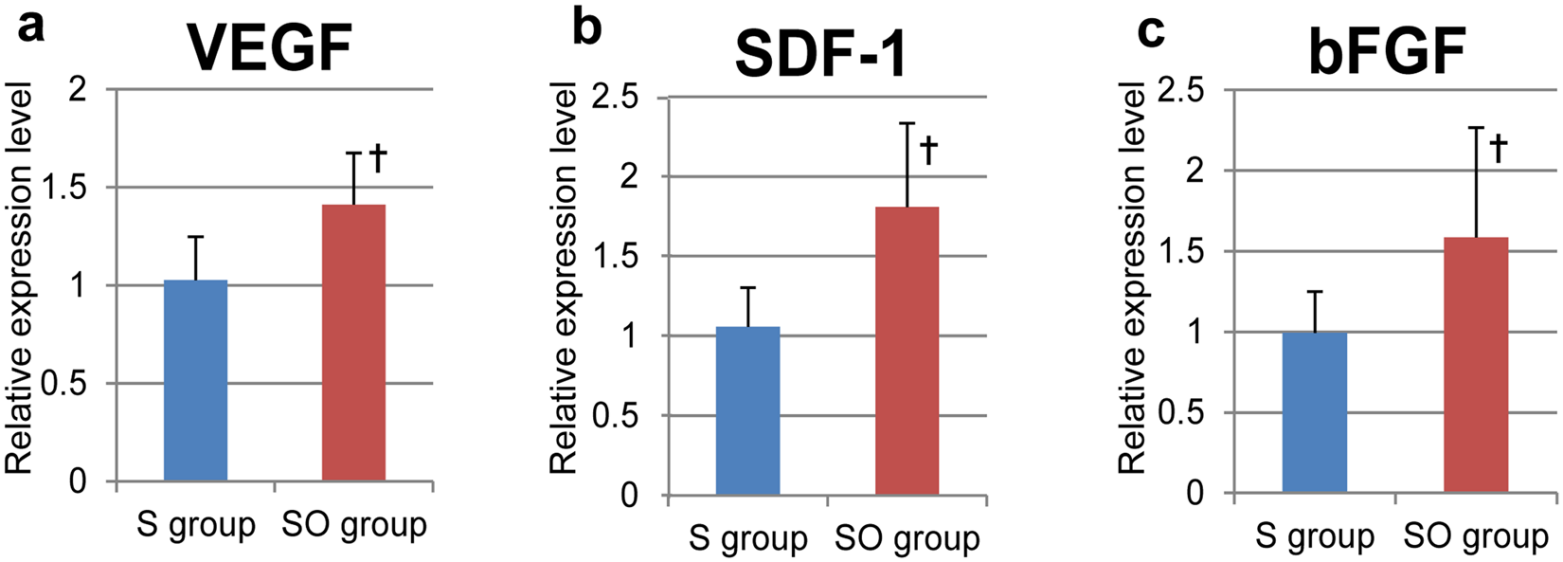
Angiogenesis-related mRNA expression in the transplanted area, as measured by RT-PCR. (**a**–**c**) Relative expression of angiogenesis-related factors at the transplanted area was significantly greater in the SO group than in the S group (**a**). VEGF, ^†^P < 0.05; (**b**) SDF-1, ^†^P < 0.05; (**c**) bFGF, ^†^p < 0.05 vs. the S group).

The combination of the omentum improved the survival rate of hiPS-CMs as shown in Fig. 5. It is expected that the omentum could improve the ischemic environment for the hiPS-CMs after transplant, and support hiPS-CMs with nutrition diffusion from its vasculatures and by secreting angiogenetic factors^12, 13^. We visualized vessels and capillaries in the sheet only and the combination therapy at 3 months after the treatment, and assessed semi-quantitatively by immunohistochemistry for vWF. In fact, capillary density in the transplanted site was significantly and markedly greater in the combination therapy (111 ± 35 units/mm^2^) than in the sheet only group (51 ± 22 units/mm^2^, P < 0.05, Fig. 6a–c). In addition, we investigated the expression level of cardioprotective and angiogenic factors in the transplanted area at 3 months after the treatment, and quantitatively assessed by real-time PCR for VEGF, SDF-1, and bFGF. Relative expression of all the factors in the transplanted site was significantly greater in the combination therapy than in the sheet only group (VEGF; 1.41 ± 0.26 vs 1.02 ± 0.22, P < 0.05, SDF-1; 1.81 ± 0.53 vs 1.06 ± 0.24, P < 0.05, bFGF; 1.59 ± 0.68 vs 0.99 ± 0.26, P < 0.05, Fig. 7a–c).

### The omentum covering supported maturation of transplanted hiPS-CMs *in vivo*

**Figure 8:**
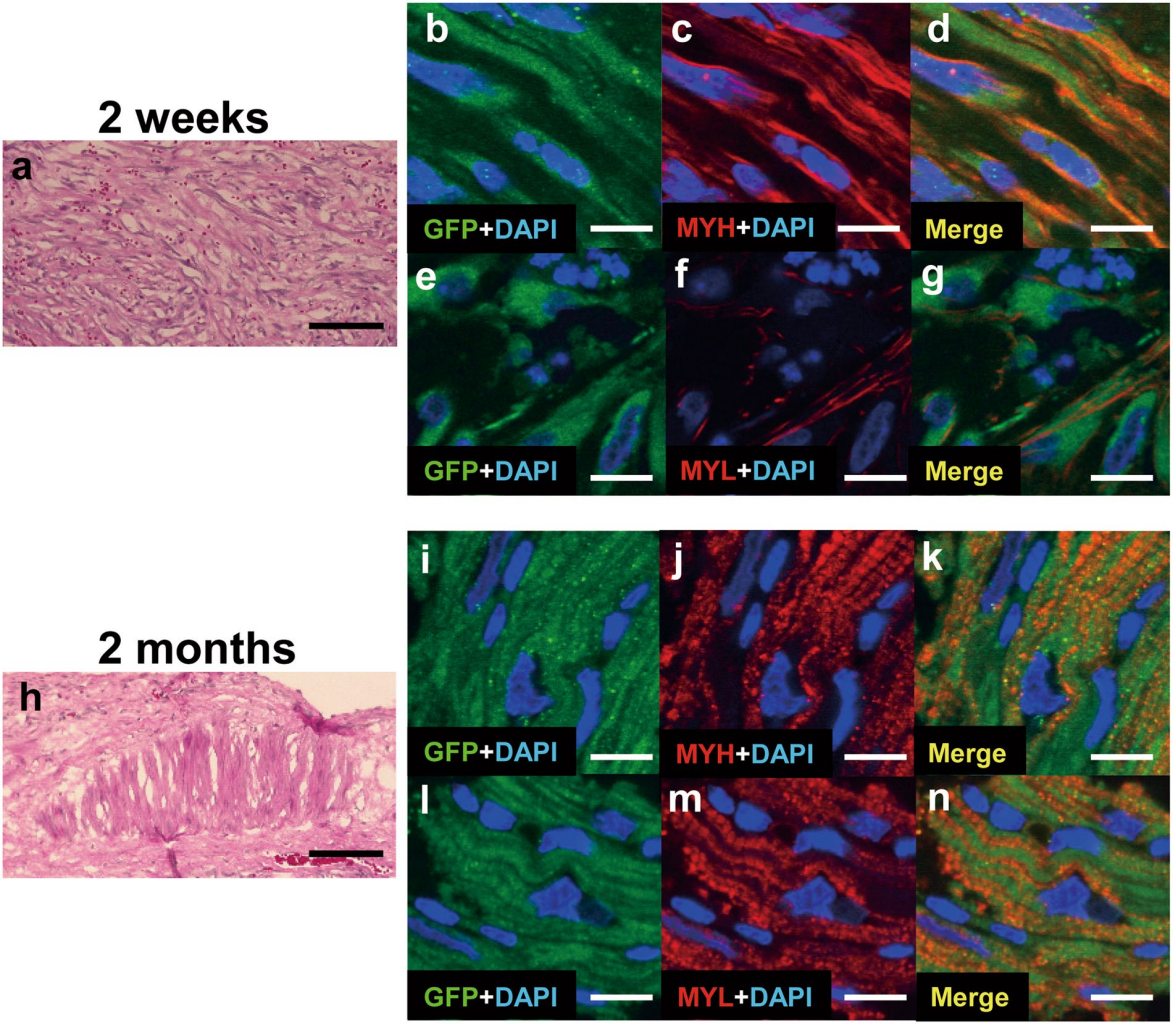
The EGFP labeled hiPS-CMs after transplantation with the omentum. (**a**–**g**) Detection of EGFP-labeled hiPS-CMs 2 weeks after transplantation; representative photomicrographs showing HE stain (**a**), GFP in green (**b**,**e**), myosin heavy chain (MYH) and myosin light chain-2 (MYL) in red (**c**,**f**). (**h**–**n**) Detection of EGFP-labeled hiPS-CMs 2 months after transplantation; representative photomicrographs showing HE stain (**h**), GFP in green (**i**,**l**), and MYH and MYL in red (**j**,**m**). The nuclei were stained with DAPI in blue (**b**–**g**,**i**–**n**). Merged images are shown in (**d**,**g**,**k**,**n**). Bar = 100 μm in (**a**,**h**), 10 μm in (**b**–**g**,**i**–**n**).

We used enhanced green fluorescent protein (EGFP)-labeled hiPS cells, which contains the sequence of EGFP driven by myosin heavy chain 6 (MYH6) promoter^21^, to create the cell-sheets, and to investigate phenotypic fate of the hiPS-CMs after transplant. We performed the cell-sheet transplant for MI induced mini-pigs with the omentum covering. Two weeks after transplantation with combination therapy, abundant EGFP and MYH double-positive cells were observed in the transplanted area (Fig. 8a–d). These cells were also positive for myosin light chain-2 (MYL, Fig. 8e–g). However, sarcomere structure was not clearly observed. In specimens obtained 2 months after transplant, double-positive cells were also detected, and a well-aligned, well-organized sarcomere structure was observed in these cells (Fig. 8h–n), which suggests that the omentum covering supported maturation of hiPS-CMs *in vivo*.

## Discussion

It is herein demonstrated that the combination of hiPS-CM cell-sheet and the omentum flap had higher and longer therapeutic effects compared with the sole hiPS-CM cell-sheet therapy in a porcine ischemic cardiomyopathy model. The number of surviving hiPS-CMs was significantly greater in the MI-induced mini-pigs with the omentum than those without it, although there was a stable reduction in the surviving cell number independent of the omentum support, as investigated by SPIO cell-labelling with CMR. The additive omentum covering to the hiPS-CM cell-sheet transplantation markedly increase the number of vessels and capillaries, associated with upregulation of VEGF, bFGF and SDF-1, at the transplanted site on the damaged heart compared to the hiPS-CM cell-sheet transplantation without the omentum. In the analysis using EGFP-labeled hiPS cells, although implanted cardiomyocytes which were transplanted 2 weeks after implantation has immature features such as poor sarcomeres or cytosols, some of the transplanted hiPS-CMs got matured and synthesized well-organized sarcomere *in vivo* in the MI-induced mini-pigs with the omentum flap 3 months after transplant.

Conditions in the host heart would influence the survival of transplanted cells after delivery, which could limit the therapeutic effects and outcomes of stem cell therapy^1–3^. Especially, the hostile ischemic environment is assumed, when the cells are delivered in the infarcted myocardium without blood supply. In our previous work, combination of cell-sheet technique with the omentum flap enhanced the cell survival after transplantation in a porcine uninjured heart^13^. In this study, the combination method also enhanced the survival of the delivered cells even in the damaged myocardium, and angiogenesis and expression of angiogenesis related factors were increased in the transplanted area. In addition, cell-sheet transplantation with the omentum flap better promoted arteriogenesis and stabilized blood vessels in ischemic myocardium along with improved coronary microcirculation physiology^22^. It was also reported that the omentum had not only angiogenic cytokines but also anti-inflammatory properties and thus facilitated tissue healing of injured tissue or organs^23^. These results suggest that transplanted cells might be provided sufficient blood supply, nutrients diffusion and anti-inflammatory effects from the omentum tissue regardless of conditions of the host myocardium. Several strategies are currently under investigation to improve cell survival, and engraftment. They include that genetic modification of the cells^24^ or implantation of the cells in scaffolds made of biocompatible matrix^25^. The pedicled omentum flap is frequently used in clinical settings, such as a treatment of mediastinitis after cardiovascular surgery. Therefore, our combination method might take an advantage of easily translating to clinical application on a standpoint of regulatory science.

Human embryonic stem (ES) cell or hiPS cell-derived cardiomyocytes structurally and electronically integrated to the host myocardium after cell transplant via needle injections in large animal studies^26–29^. We also demonstrated that iPS-CM cell-sheets integrated to the host myocardium after transplant^30^, and this is one of the most important factors in the mechanisms of stem cell derived cardiomyocyte therapy for heart failure. These transplanted cells are necessary to survive long-term with enough graft size to contribute to the host myocardium contraction. In the current study, the cell-sheet combined with the omentum flap could transfer large number of hiPS-CMs on the heart, and higher and longer therapeutic effects were demonstrated compared with sole cell-sheet transplant. Especially, with the omentum flap, loss of the transplanted cells in early phase was prevented, however, the survival rate of the transplanted cells were gradually decreased in late phase. It would be considered that chronic rejection caused the decrease in the cell survival in late phase. In recent non-human primate studies, allogenic iPS-CMs with homozygous MHC haplotype transplanted in non-human primates with heterozygous MHC haplotype by MHC-matched fashion^26, 28^. MHC-matched allogenic iPS-CM transplant with immunosuppressant drugs demonstrated high cell retaining rate 2 months after the cell transplant^31^ or no evidence of rejection during 3 month-observation period^29^. Given manufacturing and regulatory restriction, and clinical application for acute disease, allogenic cell transplant is a realistic method. Though long-term survival of a large number of the transplanted allogenic cells is still challenging, enough blood supply in early phase such as the omentum flap and optimal immune suppression including MHC-matched cell transplant and immunosuppressant drug regimen are mandatory.

Transdifferentiation of mesenchymal stem cells or cardiac stem cells into cardiomoyocytes in the heart after transplantation are controversial^32–34^. Therefore, cardiomyocytes differentiated from ES cells or iPS cells *in vitro* are promising cell sources to replenish cardiomyocyte into the failing heart. The relationship between the iPS-CMs differentiation level and the engraftment capacity was investigated in the iPS-CM transplant study using severe immunodeficiency mice (NOG mice)^21^, and the iPS-CMs purified at day 20 from initiation of differentiation had a higher engraftment rate than those purified at day 8 or day 30^21^. In addition, the day20 iPS-CMs were proliferated in early phase and maturated *in vivo* from 3 to 6 months after the transplant^21^. It was confirmed that there was the optimal differentiated stage (not too immature and not too-closely mature) in iPS-CMs for enhancing engraftment rate and maximizing therapeutic efficacy^21^, that is, *in vivo* maturation of iPS-CMs would be necessary in cell therapy using iPS-CMs. In the current study, transplanted hiPS-CMs became larger and synthesized sarcomere 2 months after transplant with the omentum compared with 2 weeks after transplant. It suggests that some of the transplanted cells got matured *in vivo*, even though xenogeneic transplant model. It might be because the omentum provides good environment to some of the transplanted cells possibly via blood supply and induction of immunologic tolerance. Given this standpoint, the omentum flap is a useful cell-delivery method in cardiac regeneration therapy.

In conclusions, the pedicle omental flap covering enhanced survival of hiPS-CMs *in vivo* and produced longer therapeutic effects at post-transplantation in a porcine ischemic cardiomyopathy model. This cell delivery system would be useful in optimizing hiPS-CM cell-sheet transplantation therapies for treating severe heart failure.

## Materials and Methods

Animal care procedures were consistent with the Guide for the Care and Use of Laboratory Animals (NIH). Experimental protocols were approved by Ethics Review Committee for Animal Experimentation of Osaka University Graduate School of Medicine (reference no. 23-002-0).

### Culture and cardiomyogenic differentiation of human iPS cells, magnetic labeling, and cell-sheet preparation

The human iPS (hiPS) cell line 201B7 that was generated using the 4 transcription factors Oct4, Sox2, Klf4, and c-Myc was used in this study^8^. Culture of the hiPS cells, formation of the embryoid bodies (EBs), and subsequent cardiomyogenic differentiation^11, 13^ and purification^18^ were carried out to generate hiPS cell-derived cardiomyocytes (hiPS-CM), as previously described in our works^11, 13^. The purified hiPS-CMs were then labeled with the superparamagnetic iron oxide (SPIO) ferucarbotran (Resovist; Bayer Pharma, Berlin, Germany), by using the hemagglutinating virus of Japan envelope (HVJ-E) vector (GenomeOne-Neo, Ishihara Sangyo, Osaka, Japan)^19, 20^. Subsequently, human mesenchymal stem cell (hMSC; Lonza, Basel, Switzerland) were seeded at a density of 5 × 10^6^ cells/dish onto 10-cm UpCell dishes (CellSeed, Tokyo, Japan), on which the SPIO-labeled hiPS-CMs were grown. The next day, the dishes were incubated at room temperature, which induced the cells to detach spontaneously to form scaffold-free hiPS-CM cell-sheets.

An enhanced green fluorescent protein (EGFP) labeled hiPS cell line was also used for phenotypic fate trucking in this study. This iPS cells contains the sequence of EGFP driven by myosin heavy chain 6 (MYH6) promoter^21^. EGFP labeled hiPS-CM cell-sheets were created with same protocols described as above.

### Flow cytometry analysis and immunocytochemistry of hiPS-CMs

Cells dissociated after hiPS cell differentiation were fixed, permeabilized, and labeled with the an antibody against cardiac troponin T (cTNT; clone 13211; Thermo Fisher Scientific, Runcorn, UK) conjugated with Alexa-488 using Zenon technology (Invitrogen, Thermo Fisher Scientific, Waltham, MA, USA), followed by analysis on a BD FACSCanto II (BD Biosciences, Franklin Lakes, NJ) with BD FACSDiva Software (BD Biosciences).

To characterize the differentiated cells from the hiPS cells, immunocytochemical analysis of sarcomeric alpha actinin (ACT; 1:100 dilution, Abcam) was performed. Antibody-labelled cells were visualized with an AlexaFluor555-conjugated goat anti-mouse secondary antibody, and cells were counterstained with DAPI, and imaged with a confocal microscope (FluoView FV10i, Olympus Japan).

### Generation of a porcine ischemic cardiomyopathy model and Study protocol

A chronic myocardial infarction (MI) model was generated by placement of an ameroid constrictor (COR-2.50-SS, Research Instruments) around the left anterior descending coronary artery in female mini-pigs (Japan Farm) weighing 20 to 25 kg^17^. Four weeks after MI induction, treatments were performed via median sternotomy under general anesthesia. All animals were immunosuppressed by daily administration of tacrolimus (0.75 mg/kg, Astellas, Tokyo, Japan), mycophenolate mofetil (500 mg, Teva Chech Industries s.r.o, Opava, Chech), and predonisolone (20 mg, Takeda Pharmaceutical Co Ltd., Osaka, Japan) daily from 5 days before transplantation until sacrifice. Twenty-eight mini-pigs were induced for MI, and mini-pigs of LVEF with 30–40% were included in this study. Total 5 mini-pigs were excluded due to early death for 3, LVEF with more than 40% for 1, and LVEF with less than 30% for 1 mini-pig. The remaining 23 mini-pigs with MI were randomly divided into 4 treatment groups: cell-sheets with the omentum flap (SO group = 7), cell-sheet only (S group = 6), the omentum flap only (O group = 5), or sham operation (Sham group = 5). The overall work flow of the study was shown in Fig. 2.

### Procedural protocols of transplant of SPIO-labeled hiPS-CM cell-sheets covered with or without the omentum flap

The procedures were described in the previous manuscript^13^. Briefly, all animals were pre-anesthetized with ketamine hydrochloride (20 mg/kg, DAIICHI SANKYO, Tokyo, Japan) and xylazine (2 mg/kg, Bayer HealthCare, Leverkusen, Germany), intubated endotracheally, and maintained by a continuous infusion of propofol (6 mg/kg/h; AstraZeneca K.K., Osaka, Japan) and vecuronium bromide (0.05 mg/kg/h; DAIICHI SANKYO). Seven SPIO-labeled hiPS-CM sheets were placed on the epicardium via the median sternotomy. In the cases of transplantation of the cell-sheet covered with the omentum flap, the omentum was mobilized to the mediastinal space via additional small upper midline laparotomy, preserving both gastroepiploic arteries and their arcade. Initially, all of 7 hiPS-CM cell-sheets were placed on the epicardium, and covered with the omentum. The omentum was stitched and fixed on the excised pericardium. The mini-pigs were then allowed to recover and were later humanely sacrificed.

We induced MI on 4 mini-pigs in addition to previous 28 mini-pigs, and transplant EGFP-labeled hiPS-CM cell-sheets with the omentum flap to investigate phenotypic fate of the transplanted hiPS-CMs. Those animals were humanely sacrificed at 2 weeks or 4 weeks after transplant for histological study (2 animals each).

### Cardiac magnetic resonance imaging

Electrocardiogram-gated cardiac magnetic resonance imaging (CMR) was performed under general anesthesia with an 8-channel cardiac coil wrapped around the chest wall^32^. CMR images were acquired on a 1.5T MR scanner (Signa EXCITE XI TwinSpeed; GE Medical Systems, Milwaukee, Wisconsin). All image analyses were performed using a specialized workstation (Virtual Place Lexus64; AZE, Tokyo, Japan). We evaluated LVEF, LVEDV, and LVESV before (as baseline), and 1 month, 2months, and 3 months after treatment. To investigate left ventricular regional wall motion, each short-axis slice was divided into 6 segments at base and mid level or 4 segments at apex level, referring a standardized left ventricular segmentation model from American Heart Association guideline^35, 36^.

To assess SPIO-labeled hiPS-CM detection, animals were imaged 1 week after transplantation. In addition, 1 animal was re-imaged at 1 month, 2 months, and 3 months after transplantation to detect SPIO-labeled hiPS-CM retention. Short-axis images with 8-mm slice thickness, including the entire heart, were obtained by pulse parameters for cardiac-gated, fast gradient-recalled echo (FGRE). The SPIO-labeled hiPS-CM hypointense area was measured using planimetry of FGRE images on the workstation (Virtual Place Lexus64). The survival proportion of hiPS-CMs was determined using the hypointense area at 1 month, 2 months, and 3 months after transplant divided by the area at 1 week after transplant as the baseline.

### Histology and immunohistochemistry

The hiPS-CM cell-sheets and the excised heart specimens were either embedded in paraffin, or in OCT compound (TissuebTek; Sakura Finetek, Torrance, CA) for frozen section. The paraffin-embedded sections were stained with hematoxylin-eosin (HE) or prussian blue that visualizes iron contents. In addition, the paraffin-embedded sections were immunolabeled with anti-human von Willebrand factor (vWF) antibody (FLEX Polyclonal Rabbit Anti-Human Von Willebrand Factor, Ready-to-Use; Dako Omnis, Glostrup, Denmark) and visualized with the horseradish peroxidase-based EnVision kit (Dako). Ten different fields were randomly selected, and the number of vWF-positive cells in each field was counted using a light microscope under high-power magnification (×200). The stained blood vessels from the 10 fields were averaged and the results expressed as vascular density (per square millimeter). The frozen sections were immunolabeled with monoclonal anti-Myosin antibody (MYH; 1:100 dilution, SIGMA, St.Louis MO), anti-Myosin Light Chain2 antibody (MYL, 1:200 dilution, Abcam, Cambridge, UK), and anti-GFP antibody (1:100 dilution, Abcam) as primary antibodies, and visualized with AlexaFluor488-conjugated goat anti-mouse (Invitrogen) and AlexaFluor555-conjugated goat anti-rabbit (Invitrogen) as secondary antibodies. Nuclei were counterstained with by 4′,6-diamidino-2-phenylindole (DAPI, Dojindo, Tokyo, Japan), and assessed using a fluorescence microscope (Biorevo BZ-9000, Keyence, Osaka, Japan) or a confocal microscope (Olympus Japan, FluoView FV10i, Tokyo, Japan).

### Real-time polymerase chain reaction

Total RNA was extracted from cardiac tissue and reverse transcribed using Omniscript reverse transcriptase (Qiagen, Hilden, Germany) with random primers (Invitrogen), and the resulting cDNA was used for real-time polymerase chain reaction (RT-PCR) with the ABI PRISM 7700 (Applied Biosystems, Stockholm, Sweden) system using pig-specific primers (Applied Biosystems) for vascular endothelial growth factor (VEGF), basic fibroblast growth factor (bFGF), and stromal derived factor-1 (SDF-1). Each sample was analyzed in triplicate for each gene studied. Data were normalized to glyceraldehyde-3-phosphate dehydrogenase (GAPDH) expression level. For relative expression analysis, the ddCT method was used, and values of the cell-sheet transplantation without the omentum were used as reference values.

### Statistical analysis

Data are expressed as means ± standard deviations (SDs). Comparisons between 2 groups were made using Welch’s t test and within-group differences were compared with the paired t test. LVEF in global cardiac function and cell survival proportion over time was assessed by repeated-measures analysis of variance (ANOVA) with group, time, and group×time interaction effects. The paired t test was used for post-hoc test in repeated-measures ANOVA. All probability values are 2-sided and values of P < 0.05 were considered to indicate statistical significance. Statistical analyses were performed using JMP 12.2 (SAS Institute, Cary, NC).

### Disclosure

Dr. Shinya Yamanaka is a scientific advisor of iPS Academia Japan without salary. Dr.Teruo Okano is a founder and a member of the board of CellSeed Inc., which has licenses for certain cell sheet-related technologies and patents from Tokyo Women’s Medical University. Dr. Teruo Okano and Dr. Tatsuya Shimizu are stakeholders of CellSeed Inc. Tokyo Women’s Medical University is receiving research funds from CellSeed Inc. No other potential conflicts of interest relevant to this article were reported.
